# A new endogean, anophthalmous species of *Parazuphium* Jeannel from Northern Morocco (Coleoptera, Carabidae), with new molecular data for the tribe Zuphiini

**DOI:** 10.3897/zookeys.103.1124

**Published:** 2011-06-10

**Authors:** Carmelo Andújar, Carles Hernando, Ignacio Ribera

**Affiliations:** 1 Departamento de Zoología y Antropología Física. Facultad de Veterinaria, Universidad de Murcia. 30071 Murcia, Spain; 2 Apartado de Correos 118, E-08911 Badalona, Barcelona, Spain; 3 Institute of Evolutionary Biology (CSIC-UPF), Passeig Maritim de la Barceloneta 37-49, 08003 Barcelona, Spain

**Keywords:** Taxonomy, new species, *Parazuphium*, identification key, endogean fauna, molecular phylogeny

## Abstract

A new species of the genus *Parazuphium* (Coleoptera, Carabidae, Zuphiini), *Parazuphium aguilerai* **sp. n.**, is described from the Tingitan peninsula in North Morocco. The only known specimen was found under a large deeply buried boulder, and belongs to an anophthalmous, depigmented and flattened species. This is the second species of blind *Parazuphium* known so far, the other being *Parazuphium feloi* Machado 1998 from a lava tube in the Canary Islands. Molecular data of the only known *Parazuphium aguilerai* sp. n. specimen are provided, and a reconstructed phylogeny based on these molecular data confirms its inclusion inside Zuphiini within Harpalinae. Identification keys to the Mediterranean and Macaronesian species of *Parazuphium* are provided.

## Introduction

The genus *Parazuphium* Latreille (Coleoptera, Dryptinae, Zuphiini) is characterized by the presence in the aedeagus of a strong ventral constriction between the basal and the distal part of the median lobe, a unique structure within the Carabidae (Antoine 1962, Mateu 1993). The genus currently includes ca. 40 species (Lorenz 2005) in the Old World and Australia (Mateu 1993), arranged in three subgenera: 1) *Neozuphium* Hürka, with a single species, *Parazuphium (Neozuphium) damascenum* (Fairmaire) (Mateu 1988), with a wide Palaearctic distribution from Central Asia and the Mediterranean to the Canaries (Hürka 1982, Mateu 1988, Machado 1992, Lencina and Serrano 1995); 2) *Austrozuphium* Baehr, with five Australasian species (Baehr 1985) and 3) *Parazuphium* s.str. Jeannel, with 32 species distributed through the Old World (Mateu 1993). The recent catalogues of Löbl and Smetana (2003) and Lorenz (2005) do not include the synonymies of *Parazuphium (Neozuphium) bactrianum* (K. Daniel & F. Daniel) and *Parazuphium (Neozuphium) novaki* (G. Müller) with *Parazuphium damascenum* (Mateu [1988]).

The species of the genus seem to be associated with deep soil or the soil crevices near rivers or temporary flooded areas (Baehr 1985, Machado 1992), and generally show a flattened habitus, some degree of depigmentation and microphthalmy. Some species are known from caves, one of them being the only previously known blind species of the genus (*Parazuphium feloi* Machado, from the Canary islands) (Machado 1998).

During an entomological expedition to North Morocco we found the single specimen of a new species of *Parazuphium*, anophthalmous and with strong modifications apparently related to its endogean habitat. Despite an attempt to collect additional material the following year no other specimen was found, possibly due to the endogean habits of this species. We describe the species here, and provide some molecular data to characterize it and to postulate its phylogenetic position among the Zuphiini for which genetic data are available (Ribera et al. 2006).

## Material and methods

The unique specimen was killed and stored in absolute ethanol in the field, and total DNA was extracted using the QIAGEN Dneasy tissue kit (Qiagen, Hilden, Germany), without destroying the external cuticle. The extracted specimen was mounted in DMHF (Dimethyl Hydantoin-Formaldehyde) on a transparent acetate label. For the morphological study and photographs we used a Zeiss Stemi 2000C Trinocular Zoom Stereomicroscope with Spot Insight Firewire digital camera and software.

### Molecular methods

Total genomic DNA for the single specimen of *Parazuphium aguilerai* sp. n. was extracted using QIAGEN Dneasy tissue kit (Qiagen, Hilden, Germany). To characterize the new species we amplified fragments of six genes, four mitochondrial and two nuclear: 3' end of cytochrome c oxidase subunit (*cox1*); a single fragment including the 3' end of the large ribosomal unit (*rrnL*), the whole tRNA-Leu gene (*trnL*) and the 5' end of the NADH dehydrogenase 1 (*nad1*); 5' end of the small ribosomal unit, 18S rRNA (*SSU*); and an internal fragment of the large ribosomal unit, 28S rRNA (*LSU*). Primers used are given in Table 1. Additionally, we extracted DNA from one specimen of *Parazuphium* cf. *baeticum* (K. and J. Daniel 1898), *Zuphium olens* Rossi 1790, *Ildobates neboti* Español 1966 and several other outgroups among Carabidae (Table 2), which were amplified for the same molecular gene fragments. PCR reactions were made using PuReTaq Ready-To-Go PCR beads (GE Healthcare, UK) and standard conditions [39 cycles using 48–50°C as annealing temperature]. New sequences have been deposited in GenBank (NCBI) with Acc. Nos JF778779-JF778845. Each individual gene matrix was aligned in MAFFT with the Q-ins-i option and default parameters. The four genes fragments were concatenated to get a final dataset of 20 taxa and 3376 bp that was employed in phylogenetic analyses. Table 2 shows taxa information, source and accession number for each DNA sequence.

**Table 1.** Primers used in the study. F, forward; R, reverse. Length refers to the aligned matrix.

Type DNA	Gene	Length	Primer	S	Primer sequence (5'- 3')	Described in:
Mitochondrial protein coding	cox1	755	Jerry (M202)	F	CAACATTTATTT-TGATTTTTTGG	(Simon et al. 1994)
			Pat (M70)	R	TCCA(A)TGCACTA-ATCTGCCATATTA	(Simon et al. 1994)
Mitochondrial ribosomal	rrnL	744	16SaR (M14)	F	CGCCTGTTTA-WCAAAAACAT	(Simon et al. 1994)
			16s-ND1a (M223)	R	GGTCCCTTACGAA-TTTGAATATATCCT	(Simon et al. 1994)
Nuclear ribosomal	LSU	1240	LS58F (D1)	F	GGGAGGAAA-AGAAACTAAC	(Ober 2002)
			LS998R (D3)	R	GCATAGTTC-ACCATCTTTC	(Ober 2002)
Nuclear ribosomal	SSU	625	5'b5.0	F	GACAACCTGGTT-GATCCTGCCAGT	(Shull et al. 2001)
				R	TAACCGCAA-CAACTTTAAT	(Shull et al. 2001)

**Table 2.** Species, locality of collection, voucher reference and accession numbers for each sequence.

Especie	Locality	Voucher	cox1	rrnL	LSU	SSU
*Laemostenus terricola*	Alicante, Spain	1583BG	JF778779	JF778796	JF778812	JF778829
*Leistus spinibarbis*	Albacete, Spain	1581BG	JF778780	JF778797	JF778813	JF778830
*Calosoma sycophanta*	Albacete, Spain	1590BG	JF778781	JF778798	JF778814	JF778831
*Carabus (Eucarabus) deyrollei*	Lugo, Spain	1553BG	JF778782	JF778799	JF778815	JF778832
*Carabus (Limnocarabaus) clathratus*	Susuz, Turkey	1600BG	JF778783	JF778800	JF778816	JF778833
*Dixus capito*	Albacete, Spain	1578BG	JF778784	N/A	JF778817	JF778834
*Pseudotrechus mutilatus*	Cádiz, Spain	36_EN	JF778785	JF778801	JF778818	JF778835
*Licinus punctatulus*	Alicante, Spain	1582BG	JF778786	JF778802	JF778819	JF778836
*Elaphropus (Tachyura) parvulus*	Pays Zaer Zaine, Morocco	64_EN	JF778787	JF778803	JF778820	JF778837
*Bembidion (Peryphus) hispanicum*	Pays Zaer Zaine, Morocco	62_EN	N/A	JF778804	JF778821	JF778838
*Bembidion (Emphanes) latiplaga*	Pays Zaer Zaine, Morocco	65_EN	JF778788	N/A	JF778822	JF778839
*Perileptus aerolatus*	Agadir, Morocco	MNHN-AF113	GQ293688	FR729593	GQ293625	GQ293503
*Trechus quadristriatus*	Huesca, Spain	MNHN-AF96	FR733908	GQ293743	GQ293619	GQ293534
*Typhloreicheia laurentii*	Sardinia, Italy	56_EN	JF778789	JF778805	JF778823	JF778840
*Dyschiriodes* sp.	Pays Zaer Zaine, Morocco	63_EN	JF778790	JF778806	JF778824	JF778841
*Nebria salina*	Albacete, Spain	1579BG	JF778791	JF778807	JF778825	JF778842
*Ildobates neboti*	Castellón, Spain	MNCN-6409	JF778792	JF778808	AM051084	DQ130051
*Drypta dentata*	Ciudad Real, Spain	98_EN	N/A	JF778809	N/A	N/A
*Zuphium olens*	Murcia, Spain	97_EN	JF778793	N/A	JF778826	JF778843
*Parazuphium cf. baeticum*	Castellón, Spain	87_EN	JF778794	JF778810	JF778827	JF778844
*Parazuphium aguilerai*	Tanger, Morocco	31_EN	JF778795	JF778811	JF778828	JF778845

### Phylogenetic methods

Bayesian phylogenetic analyses (BA) were performed with MrBayes v.3.1. (Huelsenbeck and Ronquist 2001, Ronquist and Huelsenbeck 2003), partitioning by gene with a GTR+G model applied to each partition. Two independent runs of 20,000,000 generations were conducted, each with three hot and one cold chain, whereby trees were sampled every 100 generations. Sampled trees were analysed with Tracer v.1.5 (Rambaut and Drummond 2007) and their half compact consensus tree was calculated with a burning value of 10% with node posterior probabilities used as support values, checking for an appropriate degree of convergence between chains with the effective sample size in Tracer v.1.5. MrBayes was run on-line at the freely available computational service of Bioportal (www.bioportal.uio.no). Trees were visualized in FigTree v.1.3.1 (Rambaut 2008).

## Species treatment

### Parazuphium aguilerai

Andújar, Hernando & Ribera sp. n.

urn:lsid:zoobank.org:act:B4718866-DA9E-4096-9291-A38E90FD7A0A

http://species-id.net/wiki/Parazuphium_aguilerai

Figs 1 [Fig F2] 3

#### Type locality.

Souk-Khemis-des-Anjra, Tetuan, Morocco (Fig. 4).

#### Type specimen.

 Holotype: 1♂, “MOROCCO 28-III-2008 / Souk-Khemis-des-Anjra, Tetuan / 123m N35°43'18" W5°31'23" / Andújar, Hernando, Ribera & Aguilera leg."; voucher number label “31_EN"; plus red holotype label. Type specimen mounted in DMHF in a transparent acetate label, genitalia dissected and mounted in DMHF in a separate label pinned with the specimen. Deposited in the Museu de Ciències Naturals de Barcelona (MCNB), DNA aliquots deposited in the IBE (CSIC) and Univ. Murcia (ZAFUMU col.).

#### Diagnosis.

**Figure 1.**
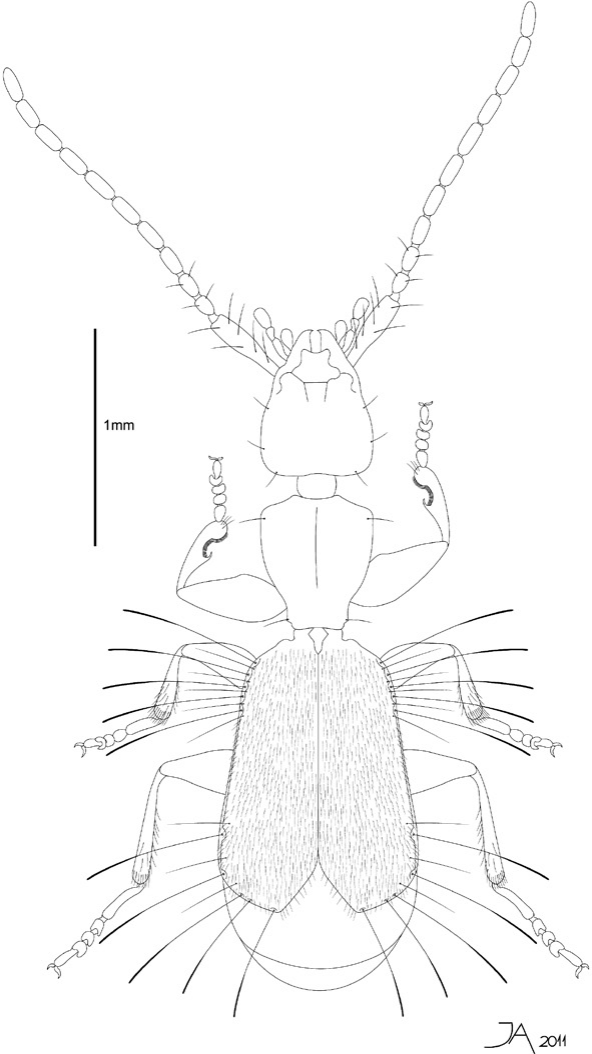
Line drawing ofhabitus of *Parazuphium aguilerai* sp. n. Total length 2.7 mm.

**Figure 2. F2:**
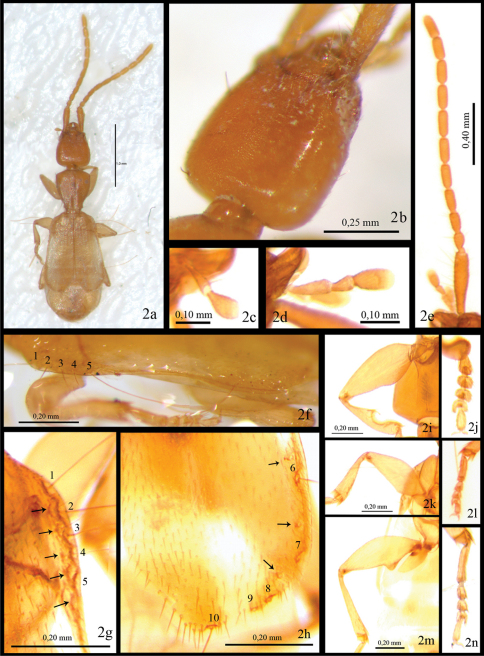
Photographic images of*Parazuphium aguilerai* sp. n. **A** whole specimen **B** head in dorso-lateral view **C** labial palpus; (d), maxillary palpus **C** antenna **F** margin of left elytron in lateral view **G** margin of right elytron, detail for anterior umbilicate setae, numbers 1 to 5 **H** margin of right elytron, detail of posterior umbilicate setae, numbers 6 to10, arrows over them point other smaller setae **I–N** details of anterior, median and posterior legs respectively.

 Total Length 2.7 mm (from apex of mandible to apex of elytra). Body depressed, flattened, light brown (Fig. 1). Eyes absent (Fig. 2b). First antennomere (0.41mm) as long as antennomeres 2–4 combined (0.37 mm) (Fig. 2e). Pronotum cordiform (Fig. 1). Elytra flat, not fully covering abdomen (Figs 1, 2a). Umbilicate lateral series of elytra with 5+5 spatuliform setae (Figs 2f-h). Apex of elytra divergent (Figs 1, 2a). Metafemora with an acute tooth on interior margin (Fig. 2m).

#### Description.

 Length of holotype: 2.7 mm. Body depressed, flattened and depigmented, light brown. Surface microreticulate, with mesh pattern regular polygonal (observed on the dried specimen) and scattered short setae.

*Head* (Fig. 1) with trapezoidal shape. No trace of eyes or ocular scars (Fig. 2b). Length of head (from apex of mandible to base) 0.63 mm; maximum width close to base (0.51 mm). Surface microreticulate, microlines deeper on sides. Neck pedunculate. With three long setae, two lateral and one basal. Appendages: antennae (Fig. 2e) with first antennomere (0.41mm) as long as total length of antennomeres 2–3-4 together (0.37 mm); second antennomere pedunculate (0.1 mm), slightly shorter than third (0.13 mm) and fourth (0.14 mm); from fifth to tenth with same length (0.16–0.17 mm); last antennomere longer (0.23mm). Antennomeres from 3° to 11° cylindrical. Labial and maxillary palpi as in Figs 2c, d.

*Pronotum* cordiform (Fig. 1), longer (0.60 mm) than wide (0.51–0.27 mm), maximum width (0.51 mm) close to anterior angles, almost double minimum width (0.27 mm), at the posterior angles. Anterior angles obtuse, rounded. Anterior margin regularly convex. Median line apparent, marked with two depressions. Two lateral setae at anterior and posterior angles. Lateral margin sinuate before posterior angles.

*Elytra* (Figs 1, 2a) flattened, short, not totally covering abdomen, wider apically (maximum width, 0.90mm, close to apex); width at humeral angle 0.65mm. Punctuation forming longitudinal series, more evident at basal third, disappearing towards apex. Entire surface with short pubescence. Anterior umbilicate series with 5 spatuliform setae (Figs 2f-g, numbers 1–5), deeply inserted in small marginal indentations, with some other minor setae over them (Fig. 2g, arrows). Posterior umbilicate series with 5 spatuliform setae, the last one just before apex (Fig. 2h, numbers 6–10), with three smaller setae over them (Fig. 2h, arrows). Margin of elytra from 5° umbilical anterior to 2° umbilical posterior seta with a marginal carina (Fig. 2f). Apices divergent (Figs 1, 2a).

*Legs*. Pro- and meso-femora dilated proximally, forming an obtuse interior angle (Figs 2i, k). Metafemora with a strong acute tooth on the interior margin (Fig. 2m). Front tibia with antennal cleaner (toilette organ), as reported in other species of the genus (Fig. 2i). Meta-tibia long and straight, with an internal spine at apex. Meso and meta tibiae with a circle of seta round the apex. Pro-tarsomeres 1–4 dilated (Fig. 2j). First meso- and meta-tarsomeres as long as 2° to 4° combined (Figs 2l, n). Fourth tarsomere cordiform. Trochanters without tooth or any other special structure.

**Figure 3.**
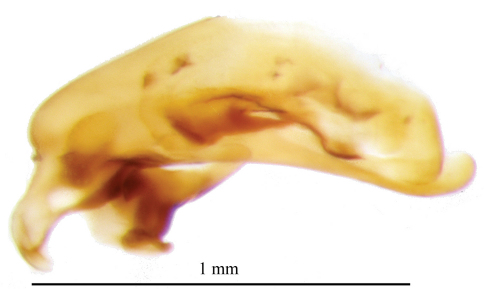
Photographic image of median lobe of *Parazuphium aguilerai* sp. n. Left lateral aspect.

*Aedeagus*. Median lobe as in Fig. 3, short and robust with a ventral constriction between the basal and the distal part as described for the genus. Basal margin arcuate, bisinuate, with the apex rounded. Internal sac with two small sclerites. Parameres asymmetric, as in other species of the genus.

#### Habitat.

**Figure 4.**
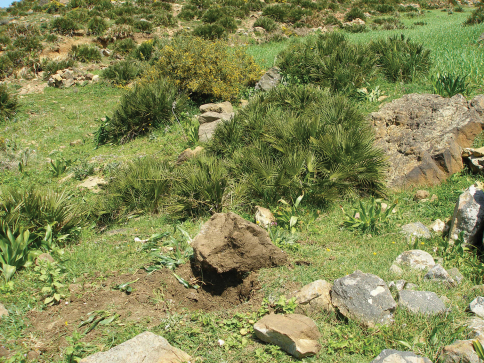
Habitat of *Parazuphium aguilerai* sp. n.

 The single known specimen of *Parazuphium aguilerai* sp. n. was found under a large, deeply buried boulder, in the humid soil on a hillside with herbaceous vegetation (*Chamaerops humilis*, *Nerium oleander* and *Pistacia lentiscus*, Fig. 4). The same sample included some endogean ants (*Leptanilla* sp, *Amblyopone* sp.) and remains of anendogean weevil, *Torneuma* sp. (Curculionidae, Cryptorhynchinae).

#### Etymology.

 The specific epithet is a Latinized eponym, genitive case, based on the name of our late friend Pedro Aguilera, who collected the specimen with us during his last trip to Morocco.

#### Recognition and comparisons.

*Parazuphium aguilerai* sp. n. can be clearly distinguished from any other species of the genus through the combinations of the following characters: lack of eyes, reduced size (2.7 mm), length and proportions of 2°, 3° and 4° antennomeres (0.1, 0.13 and 0.14mm respectively) and the presence of a tooth on metafemora. *Parazuphium feloi* from the Canary Islands is also anophthalmous, but it is larger than *Parazuphium aguilerai* sp. n. and without a tooth on the hind femora (Machado 1998). *Parazuphium ramirezi* J. and E. Vives from south Spain shows the same tooth on the metafemora, but is also larger, and with reduced eyes (Vives and Vives 1976). There are also some differences in the shape of the head and pronotum: in *Parazuphium aguilerai* sp. n. the head is more parallel-sided, the anterior angles of the pronotum are less rounded, and the anterior margin not straight.

## Identification key

Key to adults of the West Mediterranean and Macaronesian *Parazuphium* species, modified from Antoine (1962) and Hürka (1982):

**Table:** 

1	Eyeless	2
–	With eyes	3
2	Third antennal segment only slightly longer than 2nd and slightly shorter than 4th, anterior margin of pronotum trapezoidal, presence of a tooth on metafemora. Length 2.7mm. North Morocco	*Parazuphium aguilerai* sp. n.
–	Third antennal segment more than twice longer than 2nd and similar to 4th. Anterior margin of pronotum bisinuate, without tooth on metafemora. Length 4.9–5.1mm. Canary Islands	*Parazuphium feloi* Machado
3	Third antennal segment not twice as long as 2nd and distinctly shorter than 4th, legs short and robust, metaibiae curved, strongly so in male. North Africa, Middle East, Iberian Peninsula	*Parazuphium damascenum* (Fairmaire)
–	Third antennal segment at least twice as long as second and similar to 4th, metatibiae straight	4
4	Third antennal segment three times longer than 2nd. Length 7 mm. Algeria, Morocco	*Parazuphium punicum* (K. & J. Daniel)
–	Third antennal segment at most twice longer than 2nd. Length 2.8–6mm.	5
5	Eyes convex, as long as temporae, pronotum as long as wide. Length 5–5.5mm. Morocco, Tunisia	*Parazuphium vaucheri* (Vauloger)
–	Eyes flattened	6
6	Head darker than pronotum and elytra	7
–	Head concolorous with pronotum and elytra, body entirely yellowish brown	8
7	Eyes well developed, distance between hind margin of head and hind margin of eyes at most 2 times longer than diameter of eyes. Apical part of aedeagus short and robust, with slightly curved ventral margin. Length 4.5–6mm. Central and southern Europe, Turkmenistan	*Parazuphium chevrolati* (Castelnau)
–	Eyes reduced, distance between hind margin of head and hind margin of eyes at least 2.5 times longer than diameter of eyes. Apical part of aedeagus long and narrow. Morocco	*Parazuphium angusticullum* Hürka
8	Apical part of aedeagus straight, long and narrow. Length 4–5mm. Spain	*Parazuphium ramirezi* J. and E.Vives
–	Apical part of aedeagus sinuate, curved, robust and hooked. Length 3.8–5.4mm. North Africa, Italy, Spain	*Parazuphium baeticum* (K. & J. Daniel)

## Phylogenetic analysis of molecular data

**Figure 5.**
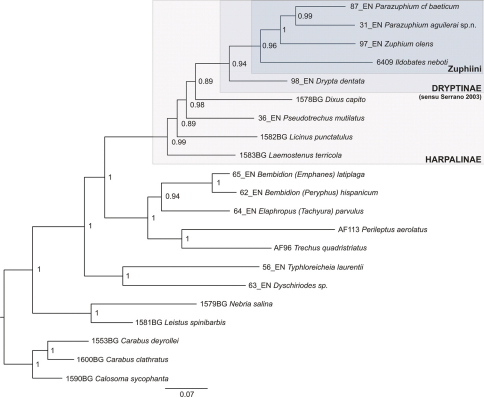
Phylogenetic tree obtained with MrBayes for the combined dataset (*cox1, rrnL, LSU, SSU*). Numbers in nodes correspond to Bayesian posterior probabilities. Zuphiini and Dryptinae (sensu Serrano 2003) indicated with bars.

The *cox1* gene fragment was aligned with no gaps, and its correct translation to amino acids confirmed. Alignment of the three ribosomal markers resulted in several gaps, which were included in the analyses as obtained from MAFFT without further modifications. Bayesian analysis reached a convergence value of 0.0005 after 20 million generations. The initial 10% saved trees were removed as a burning value and the half consensus tree was built with the “sumt" option in MrBayes v.3.1. Figure 5 represents the obtained phylogeny, were most of nodes showed very high Bayesian posterior probabilities, which are interpreted as Bayesian support.

We recovered a monophyletic Zuphiini, with the two studied species of *Parazuphium* as sisters, and sister to *Zuphium* (Fig. 5). Zuphiini was sister to *Drypta*, in a monophyletic Dryptinae (sensu Serrano 2003).

## Discussion

### Relationships of Parazuphium

The genus *Parazuphium* is currently included in subtribe Zuphiina (tribe Zuphiini), together with *Ildobates*, *Zuphium* and *Polistichus* among the Palaearctic fauna (Baehr 2003). Although the scarcity of data does not allow a comprehensive study, our molecular results support this taxonomic position, with both studied *Parazuphium* species clustered together as a sister group of *Zuphium* (Fig. 5). *Zuphium* and *Parazuphium* species are recovered as related to *Ildobates neboti*, which was found as belonging to the Zuphiini by Ribera et al. (2006). Our data confirm the close relationship of *Zuphium* and *Parazuphium*, while a more detailed phylogeny would be needed to establish the position of *Ildobates* within Zuphiini.

### Classification of Parazuphium

*Parazuphium* has traditionally been divided in three subgenera, *Neozuphium*, with only one valid species, *Parazuphium damascenum* (Mateu 1988) (note that Baehr 2003 does not include the synonymisation of *Parazuphium varum*, *Parazuphium bactrianum* and *Parazuphium novaki* by Mateu 1988); *Parazuphium* s.str., with 12 Palaearctic (Baehr 2003) and 20 Ethiopian species; and *Austrozuphium*, with five Australasian species (Baehr 1985, Lorenz 2005). The latter species are of dubious affiliation, and maybe not directly related to the Palaearctic and Ethiopian species (Mateu 1993 and pers. comm. 2008).

The subgenus *Neozuphium* was described by Hürka (1982) based on the relative length of the 2nd to 4th antennomeres and the shape of the legs, more robust and with curved tibia and enlarged femora in the males in *Neozuphium*. *Parazuphium aguilerai* sp. n. has the third antennomere only slightly longer than the 2nd and slightly shorter than the 4th (Fig. 2e), so it would agree with *Neozuphium* (species of *Parazuphium* s.str. have the 3rd antennomere double than the 2nd, and similar to the 4th, Hürka 1982, Mateu 1988). However, the shape and size of the legs do not agree with the diagnostic characters of *Neozuphium*, as the males have straight metatibia (Fig. 2m) and they are in general slender and long in comparison to *Parazuphium (Neozuphium) damascenum* (Figs 2i-n). These are, in any case, characters with dubious phylogenetic information, so instead of redefining the subgenera, or describing additional taxa, we opt to follow Serrano (2003),treating the subgenus *Neozuphium* Hürka as consubgeneric with *Parazuphium* Jeannel, and the former name as a junior synonym of the latter.

### Endogean way of life in Zuphiini

*Parazuphium aguilerai* sp. n. differs from all other known species of the genus in its clear adaptations to an endogean way of life. Other species are regularly found in soil crevices, specially among the cracks of the dried substratum of areas which are regularly inundated (Baehr 1985, Machado 1992, Lencina and Serrano 1995). These species have some modifications suggesting an adaptation to this cryptic way of life (small size, flattened body, some degree of depigmentation, microphthalmy, Jeannel 1942), but not to the extent of *Parazuphium aguilerai* sp. n., which was found in company of other typical endogean insects (*Leptanilla* sp., *Amblyopone* sp., *Torneuma* sp.) below a deeply buried large stone in a hillside. The only other anophthalmous species of the genus (*Parazuphium feloi*) was found in a cave, and it is larger and with longer appendages (Machado 1998), as is typical of cave fauna inhabiting larger open spaces. *Parazuphium aguilerai* sp. n. shares with *Parazuphium feloi*, *Parazuphium chevrolati* and *Parazuphium vaucheri* the presence of a spine in the metatibia (Machado 1998), although at the moment it is not possible to assert the phylogenetic value of this character.

## Supplementary Material

XML Treatment for Parazuphium aguilerai
